# The evolution of irradiance detection: melanopsin and the non-visual opsins

**DOI:** 10.1098/rstb.2009.0050

**Published:** 2009-10-12

**Authors:** Stuart N. Peirson, Stephanie Halford, Russell G. Foster

**Affiliations:** Nuffield Laboratory of Ophthalmology, University of Oxford, Level 5 and 6 West Wing, The John Radcliffe Hospital, Headley Way, Headington, Oxford OX3 9DU, UK

**Keywords:** circadian, melanopsin, photopigment, photoreceptor

## Abstract

Circadian rhythms are endogenous 24 h cycles that persist in the absence of external time cues. These rhythms provide an internal representation of day length and optimize physiology and behaviour to the varying demands of the solar cycle. These clocks require daily adjustment to local time and the primary time cue (zeitgeber) used by most vertebrates is the daily change in the amount of environmental light (irradiance) at dawn and dusk, a process termed photoentrainment. Attempts to understand the photoreceptor mechanisms mediating non-image-forming responses to light, such as photoentrainment, have resulted in the discovery of a remarkable array of different photoreceptors and photopigment families, all of which appear to use a basic opsin/vitamin A-based photopigment biochemistry. In non-mammalian vertebrates, specialized photoreceptors are located within the pineal complex, deep brain and dermal melanophores. There is also strong evidence in fish and amphibians for the direct photic regulation of circadian clocks in multiple tissues. By contrast, mammals possess only ocular photoreceptors. However, in addition to the image-forming rods and cones of the retina, there exists a third photoreceptor system based on a subset of melanopsin-expressing photosensitive retinal ganglion cells (pRGCs). In this review, we discuss the range of vertebrate photoreceptors and their opsin photopigments, describe the melanopsin/pRGC system in some detail and then finally consider the molecular evolution and sensory ecology of these non-image-forming photoreceptor systems.

## 1. Introduction

The 24 h cycle of light and dark caused by the rotation of the Earth produces dramatic but predictable changes in the light environment. Instead of passively responding to these changes, organisms have evolved an endogenous representation of the 24 h day—a circadian timing system. These circadian clocks set the time or phase at which physiological and behavioural events occur with respect to the external 24 h environmental cycle. In this way, change can be anticipated, and physiology is optimized to the varying demands of night and day. This allows the organism to exploit the changed conditions as soon as they take place; avoiding the time lost in physiological and behavioural adjustments. However, the circadian system will only provide a selective advantage if biological time remains synchronized (entrained) to environmental time. Thus, the circadian oscillator requires a daily synchronization with the external environment via time cues termed zeitgebers (time givers) (Aschoff 1984; Pittendrigh 1993). The systematic daily change in the gross amount of environmental light (irradiance) at dawn or dusk provides the primary indicator of the time of day. As a result, most organisms have evolved to use the twilight transition as their main zeitgeber to adjust circadian time to local time. This process is termed photoentrainment (Roenneberg & Foster 1997).

The study of the photoreceptors mediating irradiance-detection tasks such as photoentrainment has led to the identification of a range of vertebrate opsins, and perhaps most remarkably, the identification of a novel photoreceptor system within the mammalian retina, a subset of retinal ganglion cells (RGCs) that express the photopigment melanopsin (*Opn4*). In this review, we will consider the range of vertebrate photoreceptors and their opsin photopigments and then provide an overview of the melanopsin/photosensitive retinal ganglion cell (pRGC) system in detail. Finally, we will discuss the molecular evolution of the opsin photopigments and consider the different selective pressures acting upon visual pigments and non-image-forming photopigments in general.

## 2. Vertebrate photoreceptors

**Figure 1.**
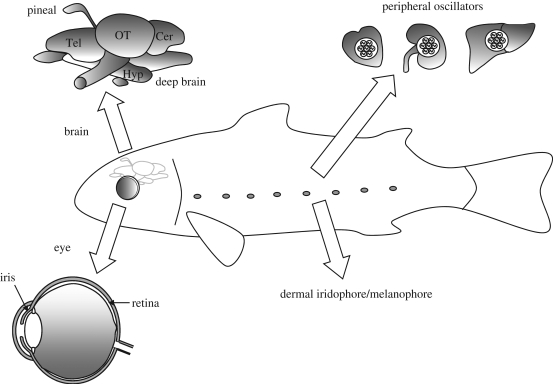
Photoreceptive sites in the vertebrates. As well as the classical photoreceptors within the retina of the lateral eye, direct photoreception in the isolated iris has also been described. In non-mammalian species the pineal complex also contains photoreceptors, and deep brain photoreceptors may also occur. Dermal photoreception has been described in amphibians and fish. Finally, in the zebrafish peripheral tissues have been shown to be able to entrain their molecular oscillators directly to light. See text for further details.

In mammals, both visual and non-visual photoreception is ocular, and enucleation abolishes all responses to light (Nelson & Zucker 1981; Foster *et al*. 1991). By contrast, non-mammalian vertebrates possess a wide range of photoreceptive sites, including the pineal complex, deep-brain photoreceptors and dermal photoreceptors (Shand & Foster 1999). As well as being anatomically diverse (figure 1), these photoreceptors mediate many different aspects of physiology and behaviour. Identifying the extraretinal opsin photopigments that underlie these responses in non-mammalian photoreceptors has a long and fairly complex history. Many immunocytochemical studies were undertaken on these tissues, using a wide range of antibodies raised to different retinal or visual pigment preparations (Shand & Foster 1999). However, as the epitopes/specificities of many of these antibodies were not known, it was difficult to make any definitive conclusions regarding the molecular identity of the molecules labelled. The ambiguous terms ‘rod-like’ or ‘cone-like’ were frequently used to describe such immunolabelling. The molecular characterization of the extraretinal photopigments became further blurred with the discovery of multiple new opsin families, quite different from the rod and cone opsins. Parallel studies on mammals also produced results that were initially difficult to interpret. Although it was clear that mammals lack extraocular photoreceptors (Nelson & Zucker 1981; Foster *et al*. 1991), mice lacking rod and cone photoreceptors could still regulate multiple physiological responses to light (Freedman *et al*. 1999; Lucas *et al*. 1999). Further, these responses were clearly being mediated by an opsin/vitamin A-based photopigment system (Lucas *et al*. 2001). Yet until recently the molecular identity of this photoreceptor system remained unknown. In the past decade much new information has emerged regarding the location and function of the vertebrate non-rod, non-cone photoreceptor systems. Here in §2, we take the opportunity to summarize some of these findings, and in §3 we discuss the photopigment biochemistry in detail.

### (a) Lateral eyes

The lateral eyes are the most familiar photoreceptive site in vertebrates. The classical photoreceptors of the vertebrate retina consist of the rods and cones. Rods mediate scotopic (dim light) vision, providing low-resolution but high sensitivity, whereas cones are involved in photopic (bright light) vision, and enable high-resolution colour vision/contrast detection. Rod and cone light detection is characterized by rapid and transient electrical responses. The graded potentials from these receptors are processed by inner retinal neurons prior to advanced visual processing in the brain. Light information reaches the visual centres of the brain via topographically mapped axons of the RGCs that form the optic nerve (Rodieck 1998). But in addition to the ‘classical’ photoreceptors of the outer retina, other retinal cells are now also known to be capable of responding to light. A subset of RGCs (approx. 1% in the mouse) expresses the photopigment melanopsin (*Opn4*; Hattar *et al*. 2002) and are capable of responding to light directly (Berson *et al*. 2002; Sekaran *et al*. 2003). The identification of these pRGCs is discussed in more detail in §4.

In addition to melanopsin-based pRGCs, the teleost retina (and perhaps other non-mammalian retinae) possesses photosensitive horizontal cells. In the cyprinid retina of the roach (*Rutilus rutilus*), a subtype of the horizontal cell, termed HC-RSD, expresses both melanopsin and vertebrate ancient (VA) opsin and shows depolarizing responses to light that are maximally sensitive to approximately 477 nm. These cells have longer integration times than rods or cones and maintain their responses when classical photoreceptor inputs are saturated by background light (Jenkins *et al*. 2003). These cells may signal environmental irradiance as well as modulating rod and cone outputs.

### (b) Pineal

Perhaps the best known photoreceptive site outside the retina is the pineal organ (*epiphysis cerebri*). Here we will use the term pineal complex to refer to the intracranial pineal proper as well as the parapineal and the extracranial ‘third’ eyes found in tuatara (*Sphenadon punctatus*, Rhynchocephalia), some lizards (Squamata) and frogs (Anura). The intracranial parapineal organ only occurs in some species of fish, and remarkably little is known about the physiological functions of this enigmatic organ (Vollrath 1981; Shand & Foster 1999). The extracranial third eyes can be further subdivided into the frontal organs (or *Stirnorgan*) of anuran amphibians and the parietal eyes found in lizards. The parietal eye shows remarkably structural similarity to the lateral eyes, with a transparent cornea and lens (Shand & Foster 1999).

Embryologically, the pineal complex is derived from an evagination of the dorsal diencephalon, similar to the retina, and in non-mammalian vertebrates is located near the surface of the brain (Vollrath 1981). In teleost and cyclostome species there is often a translucent window or area of reduced pigmentation overlying the pineal, allowing approximately 10 per cent of the incident light to reach the pineal. In amphibians, reptiles and birds, such a pineal window is less apparent or absent. Despite this, a considerable amount of light is still able to penetrate the overlying tissues, amounting to 0.1 per cent−0.3 per cent of the incident light (Dodt & Meissl 1982). In all non-mammalian vertebrates, the pineal complex is photoreceptive, and the predominant cell type is photoreceptor-like in appearance. In mammals, the pineal organ expresses many elements of the phototransduction cascade (Korf *et al*. 1985*a*,*b*), but lacks photosensitivity and appears exclusively secretory (Foster *et al*. 1989, 2003). The pineal organ is the primary source of the neurohormone melatonin, which is synthesized in the dark phase of the light/dark cycle, and acts as a signal of darkness to regulate circadian rhythms and photoperiodic responses (Arendt 1998; Korf *et al*. 1998). Melatonin synthesis is locally regulated by light at the level of the pineal in non-mammalian vertebrates, but in mammals photic information reaches the pineal via a multisynaptic pathway via the retinohypothalamic tract (RHT) and the superior cervical ganglion of the sympathetic nervous system (Korf & Moller 1984; Meissl 1997).

A range of opsins has been detected in the pineal complex of vertebrates (Shand & Foster 1999). One of the first extraretinal opsins to be identified, Pinopsin (P-opsin), was isolated from the avian pineal (Okano *et al*. 1994; Max *et al*. 1995). In the teleost pineal, a range of rod and cone opsins along with VA-opsin are expressed (Philp *et al*. 2000*b*). However, it appears that the predominant opsin in the fish pineal is a rod-like opsin (exo-rhodopsin/extraretinal rod-like opsin) which differs from that found in the lateral eyes (Mano *et al*. 1999; Philp *et al*. 2000*a*). Despite the lack of information about the structure and function of the parapineal organ a novel opsin photopigment, parapinopsin, has been isolated specifically from the parapineal of the channel catfish (*Ictalarus punctatus*) (Blackshaw & Snyder 1997) as well as from the lamprey pineal (Koyanagi *et al*. 2004). Most recently, studies on the parietal eye have identified the expression of two opsins within the same cell, a blue-sensitive pinopsin and a novel green-sensitive opsin named parietopsin (Su *et al*. 2006).

### (c) Deep brain

Deep-brain photoreceptors were first described following studies by Karl von Frisch in 1911 on blinded and pinealectomized European minnows (*Phoxinus phoxinus*). These fish still demonstrated colour changes in response to light, leading to the suggestion of ‘deep-diencephalic photoreceptors’ (von Frisch 1911). Similarly, studies in blinded pinealectomized European eels (*Anguilla anguilla*) by van Veen *et al*. (1976) showed that deep-brain photoreceptors mediate photoentrainment as well as negative phototaxis. The photoperiodic response in birds, whereby gonadal growth is regulated by day length, is also mediated by a deep-brain photoreceptor rather than by the pineal complex or lateral eyes (Benoit 1964). Action spectroscopy provided a clue as to the molecular identity of these photoreceptors. An absorption corrected action spectrum for photoperiodic induction in the Japanese quail described an opsin/vitamin A-based photopigment with a *λ*_max_ at 492 nm. A recent reanalysis of the original data suggests that the *λ*_max_ may be closer to 483 nm (S. N. Peirson & R. G. Foster 2008, unpublished data). Although this action spectrum inferred the biochemistry of the photopigment, the precise molecular identity still remains unresolved. Attempts to characterize these photoreceptors have involved the use of immunocytochemical techniques employing antibodies raised against rod and cone photopigment opsins or other elements of the phototransduction cascade. Such approaches either failed to localize opsins within the avian hypothalamus or produced ambiguous results owing to the use of unknown epitopes (Silver *et al*. 1988).

### (d) Iris

Light striking the isolated iris has been reported to produce rapid sphincter pupillae constriction in several non-mammalian species of vertebrate. For example, an opsin/vitamin A type action spectrum has been described from the isolated frog iris (Barr & Alpern 1963) and eel iris (Selinger 1962). In the isolated chick iris, there are marked and rapid responses to light (Tu *et al*. 2004). In some mammalian species, there have also been reports of an extremely gradual pupil constriction (occurring over at least 20 s to very bright stimuli) that survives both isolation of the iris from the eye and application of atropine (Bito & Turansky 1975; Lau *et al*. 1992). In all cases, a definitive photopigment characterization for iris photosensitivity is lacking, although in *Xenopus laevis* melanopsin has been implicated (Provencio *et al*. 1998).

### (e) Dermal

Photoreception by dermal cells mediates colour changes in chromatophores and iridophores. Dermal photoreception has also been linked to the triggering of locomotor activity (Wolken & Mogus 1979; Shand & Foster 1999). Dermal chromatophores are photosensitive in many vertebrates, regulating the aggregation and dispersal of pigment granules within these cells (Weber 1983). Melanopsin, the photopigment of retinal pRGCs, was first isolated from *Xenopus laevis* melanophores (Provencio *et al*. 1998) and appears to be the photopigment mediating the pigment dispersal responses within dermal melanophores (Isoldi *et al*. 2005).

### (f) Tissue photoreception

Perhaps the most surprising result in recent years has been the discovery that the circadian clocks located within the peripheral organs (such as the heart and kidney) of zebrafish can be entrained to light after being isolated and maintained *in vitro* (Whitmore *et al*. 2000). The photopigment(s) mediating these responses remain poorly understood, although the widespread expression pattern of teleost multiple tissue (TMT) opsin (see below) makes this opsin a strong candidate.

## 3. Opsin photopigments

**Figure 2.**
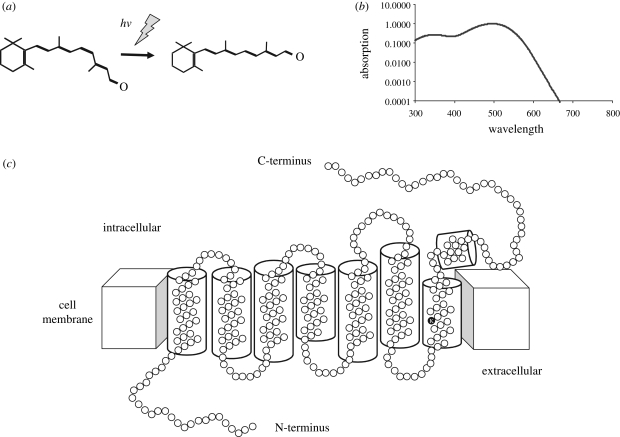
Structure and function of vertebrate photopigments. Vertebrate photopigments consist of an isomer of vitamin A, retinaldehyde, bound to an opsin protein. (*a*) The primary step in phototransduction is the absorption of a photon of light (*hv*) by the 11-*cis* isomer of retinal resulting in isomerization to the all-*trans* form. (*b*) All vitamin A/opsin-based photopigments have a characteristic absorption spectrum which can be used as a ‘spectral fingerprint’ to determine the photopigment mediating a given biological response. (*c*) Opsins consist of a single polypeptide chain forming seven α-helical transmembrane regions connected by cytoplasmic and extracellular loops. The intracellular domains mediate G-protein interactions. The retinal binding site (K) is indicated in the 7th transmembrane domain. Structure based on that of Palczewski *et al*. 2000.

All vertebrate photoreceptors identified to date use an opsin/vitamin A-based photopigment. These photopigments consist of an opsin protein bound to a vitamin-A chromophore. In most terrestrial and marine vertebrates, this chromophore is 11-*cis*-retinaldehyde (A_1_), while in many freshwater vertebrates the chromophore is based upon 11-*cis*-3-dehydroretinal (A_2_) (Knowles & Dartnall 1977). The first stage of light detection involves the absorption of a photon by the retinal chromophore and the photoisomerization of this molecule to the all-*trans* state (figure 2*a*). The conformational change of the chromophore allows the opsin to interact with a G-protein (transducin) and triggers the phototransduction cascade, ultimately giving rise to a change in receptor membrane potential (Hargrave & McDowell 1992; Okada *et al*. 2001; Pepe 2001; Shichida & Matsuyama 2009). All vitamin A-based photopigments have a characteristic absorption spectrum. This means that although the maximal sensitivity (*λ*_max_) of the pigment may vary widely across the visible spectrum (ultraviolet at 360 nm to deep red at 620 nm), all these pigments have the same characteristic shape, a bell-shaped curve (figure 2*b*) (Dartnall 1953).

Opsins are members of the superfamily of G-protein-coupled receptors (GPCR) which function through the activation of a guanine nucleotide binding protein (G-protein) and an effector enzyme. All opsins consist of a single polypeptide chain of 340–500 amino acids that form seven α-helical transmembrane regions connected by cytoplasmic and extracellular loops (figure 2*c*). These seven α-helices form a bundle within the membrane, creating a hollow cavity on the extracellular side that serves as a binding site for the chromophore, retinal. There are also several other features usually present in an opsin: the retinal attachment site is a lysine residue (at position 296 numbered as in bovine rod opsin) located in the seventh transmembrane domain which binds the chromophore via a protonated Schiff base linkage; a glutamate counterion at position 113 in the third transmembrane domain; a glutamate, arginine and tyrosine (ERY, 134–136) tripeptide for G-protein interaction; and cysteines (C) at positions 110 and 187 for disulfide bridge (Palczewski *et al*. 2000).

**Figure 3.**
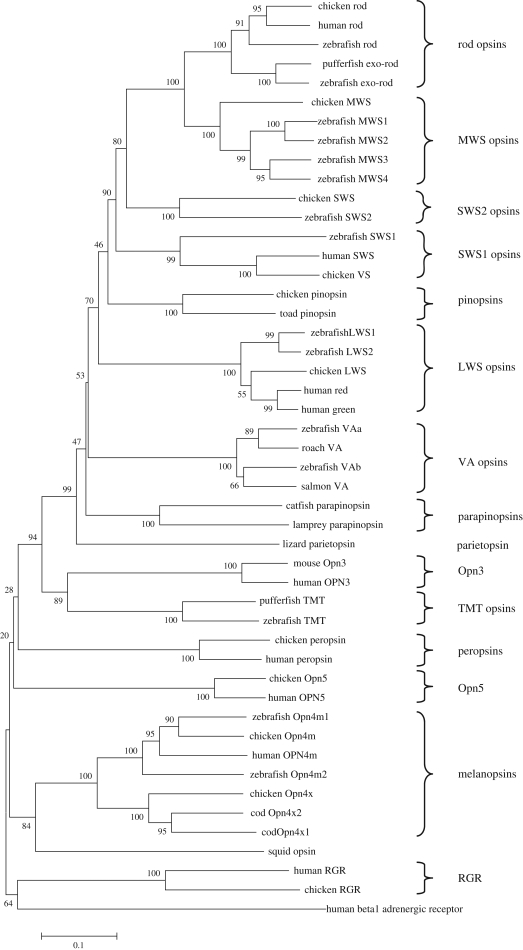
Phylogenetic tree showing the relationship of the various classes of vertebrate opsins. The entire amino acid sequences were aligned using ClustalW (Higgins *et al*. 1996) and the tree was generated by the neighbour joining method (Saitou & Nei 1987) using the MEGA4 program (Tamura *et al*. 2007). Branch confidence levels (% based on 1000 bootstrap replicates) are marked. Scale bar indicates substitutions per site. The human beta 1 adrenergic receptor was used as an outgroup. The major classes of the vertebrate opsins are indicated by parentheses on the right-hand side. The analysis reveals that the exo-rod opsins are a duplication of the rod opsins and that pinopsin has arisen by a duplication of the cone opsins. To date there is only one sequence for parietopsin (see text for details). Opn3 and TMT, both expressed in multiple tissues, seem to share a common ancestor. The Opn4 sequences now quite clearly consist of two families, the mammalian-like ‘m’ form and the *Xenopus*-like ‘x’ form. Interestingly the opsin from the invertebrate squid also included in the analysis clades with the melanopsin sequences, adding credence to the argument that melanopsin is ‘invertebrate’-like. Finally RGR is the least similar to the visual opsins. Accession numbers: Chicken: rod D00702; LWS M62903; MWS M92038; SWS M92037; versus M92039; RGR AY339627; peropsin AY339626; pinopsin U15762; Opn4m AY036061; Opn4x AY882944; Opn5 XM_001130743. Human: rod NM_000539; red NM_020061; green NM_000513; SWS NM_001708; RGR NM_002921; peropsin NM_006583; OPN3 NM_014322; OPN4M NM_033282; OPN5 NM_181744; beta 1 adrenergic receptor NM_000684. Zebrafish: rod NM_131084; LWS1, NM_001002443; LWS2, NM_131175; MWS1, NM_131253; MWS2, NM_182891; MWS3, NM_182892; MWS4, NM_131254; SWS1, NM_131319; SWS2, NM_131192; exo-rod, AB025312; VA1, AB035276; VA2, AY996588; TMT, AF349947; Opn4m1, AY882945; Opn4m2, AY078161. Pufferfish: exo-rod, AF201472; TMT, AF402774. Salmon VA AF001499. Roach VA AY116411. Catfish parapinopsin AF028014. Lamprey parapinopsin AB116380. Lizard parietopsin DQ100320. Toad pinopsin AF200433. Mouse Opn3 NM_010098. Cod Opn4x1 AF385823. Opn4x2 AY126448. Squid opsin, P09241.

A wide range of opsins have been identified within the vertebrates, with currently 15 distinct gene families (table 1). These are described in detail below and a phylogenetic analysis to illustrate their molecular evolution is shown in figure 3.

**Table 1.** Vertebrate opsin subgroups. Table showing the amino acid identity (%) across the ‘core’ region of representatives of the various vertebrate opsin classes. The core region is defined by residues 34–306 of the bovine rod opsin model of Palczewski *et al*., (2000). The chicken sequences were used where available, other species are stated below. Accession numbers: rod opsin D00702; LWS M62903; MWS M92038; SWS M92037; versus M92039; RGR AY339627; peropsin AY339626; pinopsin U15762; Opn4m AY036061; Opn4x AY882944; Opn5 XM_001130743; catfish parapinopsin AF028014; lizard parietopsin, DQ100320; zebrafish VA1 AB035276; zebrafish VA2, AY996588; pufferfish exo-rod AF201472; human OPN3 NM_014 322; pufferfish TMT AF402774. Abbreviations as follows: exo, exo-rod; per, peropsin; P, pinopsin; PP, parapinopsin; par, parietopsin.

opsin	rod	LWS	MWS	SWS1	SWS2	RGR	per	P	PP	par	VAa	VAb	exo	Opn3	TMT	Opn4m	Opn4x	Opn5
rod opsin	—																	
LWS opsin	41	—																
MWS opsin	69	42	—															
SWS1 opsin	45	40	49	—														
SWS2 opsin	51	41	53	50	—													
RGR	28	22	21	21	23	—												
peropsin	27	24	23	21	24	24	—											
pinopsin	46	49	48	47	51	24	26	—										
parapinopsin	40	40	40	40	41	24	28	48	—									
parietopsin	36	35	35	30	37	23	27	42	42	—								
VAa	38	42	40	41	43	22	27	43	43	41	—							
VAb	37	40	40	41	41	20	26	45	46	40	80	—						
exo-rod	78	42	66	46	49	27	27	48	40	35	39	36	—					
Opn3	30	26	31	27	32	25	29	30	29	29	31	30	30	—				
TMT	33	34	34	35	36	22	29	38	39	37	36	34	30	41	—			
Opn4m	30	30	30	32	27	26	27	28	29	31	29	29	30	27	34	—		
Opn4x	29	33	29	28	26	27	29	29	28	30	28	28	32	31	34	59	—	
Opn5	25	24	26	20	25	26	30	26	25	26	26	24	25	28	29	33	29	—

### (a) Visual rod/cone opsins (Opn1, Opn2)

The well-characterized photoreceptors of the vertebrate eye are the rods and cones of the outer retina. These cells contain an opsin located in the lamellae of their outer segments which were named after the photoreceptor class in which they have been found, although they have now been given gene symbols, *Opn1* for cone opsins and *Opn2* for rod opsin. Bovine rod opsin was the first opsin to be cloned and sequenced (Nathans & Hogness 1983) and is still the only vertebrate opsin to have its crystal structure determined (Palczewski *et al*. 2000). There are four classes of cone opsins, long-wave sensitive (LWS) with a *λ*_max_ between 500 and 620 nm; medium wave sensitive (MWS) *λ*_max_ of 480–520 nm; ultraviolet/violet sensitive (SWS1) *λ*_max_ 355–435 nm and short-wave sensitive (SWS2) *λ*_max_ 415–470 nm. The various cone classes show 40–50% amino acid identity to each other (table 1). The SWS2 and MWS opsins have been lost in the mammalian lineage (Bowmaker & Hunt 1999).

### (b) RPE opsins (RGR, peropsin)

Two opsin classes have been shown to be expressed in the retinal pigment epithelium (RPE), retinal G-protein-coupled receptor (RGR) and peropsin. RGR was originally identified from a bovine RPE library and although it contains the lysine required for chromophore attachment it has a histidine at the position of the potential counterion (Jiang *et al*. 1993). This sequence and its expression in the RPE and the Müller cells led to suggestions that it may function as an 11-*cis*-retinal transporter or an all-*trans* retinal photoisomerase (Jiang *et al*. 1993; Pandey *et al*. 1994). Although further studies showed that RGR will bind all-*trans*-retinal and generate 11-*cis*-retinal in the RPE (Hao & Fong 1996), more recent work by the same group has shown that the isomerized 11-*cis*-retinal does not readily dissociate from RGR, so it does not make a significant contribution to the pool of 11-*cis*-retinal (Hao & Fong 1999).

Peropsin, also called RPE-derived rhodopsin homologue, was isolated in 1997 (Sun *et al*. 1997) and only shows approximately 27 per cent identity to the visual opsins. It is localized to the RPE and there is some evidence that it shares a common ancestor with RGR. This has led to the speculation that it may also function as a retinal isomerase (Bellingham *et al*. 2003). Melanopsin has also been shown to be expressed in the RPE (Peirson *et al*. 2004), although whether this opsin forms a functional photopigment in the RPE remains unclear. Further work is required to clarify the role of these RPE opsins.

### (c) Pineal opsins (pinopsin, parapinopsin, parietopsin, exo-rod)

As discussed in §2*b*, the pineal complex is photosensitive in non-mammalian vertebrates and multiple opsins have been isolated from this part of the brain.

#### (i) Pinopsin

Pinopsin was the first extraretinal opsin to be cloned and was isolated from the pineal gland of the chicken (Okano *et al*. 1994; Max *et al*. 1995). It showed 43–48% amino acid identity to the vertebrate visual opsins (table 1) and in chicken is expressed exclusively in the pineal. Several groups have reported the *in vitro* expression and reconstitution of pinopsin with 11-*cis*-retinal. All report the formation of a blue-sensitive pigment, but with slightly different *λ*_max_ values: approximately 470 nm (Okano *et al*. 1994); approximately 462 nm (Max *et al*. 1998); and approximately 460 nm (Nakamura *et al*. 1999). Pinopsin has also been identified from the Reptilia (Kawamura & Yokoyama 1997) and Amphibia (Yoshikawa *et al*. 1998). Pinopsin has been localized to the anterior preoptic nucleus of the hypothalamus in the toad (Yoshikawa *et al*. 1998) and interestingly in both the retina and pineal of a diurnal gecko (Taniguchi *et al*. 2001). By contrast, a study on the Ruin lizards (*Podarcis sicula*) suggested that pinopsin was only expressed in the pineal complex (Frigato *et al*. 2006). Pinopsin orthologues have not, to date, been isolated from either fish or mammals.

#### (ii) Parapinopsin

Despite the lack of information about the structure and function of the teleost parapineal, a novel opsin photopigment—parapinopsin—has been isolated from this organ. Until recently there was only one reported sequence for parapinopsin isolated from the channel catfish (*Ictalurus punctatus*; Blackshaw & Snyder 1997). The sequence shows 40 per cent identity to other vertebrate opsins (table 1) and is expressed in a majority of parapinealocytes and a subset of pineal cells. However, a homologue of parapinopsin was isolated from the lamprey pineal complex and appears to form a bi-stable photopigment (Koyanagi *et al*. 2004). *In situ* hybridization showed that lamprey parapinopsin is expressed in the photoreceptor cells located in the dorsal region of the pineal and parapineal organs. The authors also demonstrated that lamprey parapinopsin photopigment has a *λ*_max_ at 370 nm and that UV light causes *cis*–*trans* isomerization of its retinal chromophore, forming a stable photoproduct with *λ*_max_ at 515 nm (Koyanagi *et al*. 2004). The authors of this paper also report the isolation of parapinopsin sequences from the rainbow trout and the clawed frog which exhibit 61 and 71 per cent amino acid identity, respectively, to the lamprey sequence (Koyanagi *et al*. 2004).

#### (iii) Parietopsin

Most recently, studies on another photoreceptive structure, the parietal eye of a lizard, have identified expression of two opsins within the same photoreceptor, a blue-sensitive pinopsin and a novel green-sensitive opsin named parietopsin (Su *et al*. 2006). These findings are consistent with the observation that the parietal eye photoreceptors have two antagonistic light signalling pathways, a hyperpolarizing pathway maximally sensitive to blue light and a depolarizing pathway maximally sensitive to green light (Solessio & Engbretson 1993). Parietopsin showed the highest degree of amino acid identity (approx. 40%) to parapinopsin (table 1) (Su *et al*. 2006).

#### (iv) Exo-rod opsin

Vigh-Teichmann and colleagues first reported the presence of opsin immunoreactivity in the teleost pineal in the early 1980s (Vigh-Teichmann *et al*. 1982, 1983). However, it was not until the independent isolation of a rod-like opsin from the pineal of the zebrafish (Mano *et al*. 1999) and from the pufferfish and Atlantic salmon (Philp *et al*. 2000*a*) that the molecular identity of this opsin was elucidated. Exo-rod opsins are 74 per cent identical to the retinal rod opsin from the same species suggesting that they diverged early in the teleost lineage (Philp *et al*. 2000*a*). Their expression is restricted to the pineal gland and their exact function remains unknown.

### (d) VA opsin

VA opsin was first described in the Atlantic salmon (*Salmo salar*) (Soni & Foster 1997) and was subsequently isolated from several other teleost fish: zebrafish (*Danio rerio*) (Kojima *et al*. 2000), the common carp (*Cyprinus carpio*) (Moutsaki *et al*. 2000), a smelt fish (*Plecoglossus altivelis*) (Minamoto & Shimizu 2002) and roach (*Rutilus rutilus*) (Jenkins *et al*. 2003). VA opsins show 37–41% identity with the rod and cone opsins and approximately 43 per cent identity to other non-visual opsins such as pinopsin (table 1). Phylogenetic analysis suggests that the VA opsins diverged from a common ancestor before the other known opsin families (Soni & Foster 1997). Lamprey (*Petromyzon marinus*) pinopsin originally described by Yokoyama and Zhang (Yokoyama & Zhang 1997) is now considered to be a member of the VA family (Moutsaki *et al*. 2000; Bellingham & Foster 2002).

Functional studies demonstrated that salmon VA opsin can form a photopigment with a *λ*_max_ between 460 and 480 nm when expressed *in vitro* and reconstituted with 11-*cis*-retinal (Soni *et al*. 1998). Significantly VA opsin was shown to be expressed in a subset of horizontal cells and retinal ganglion cells (Soni *et al*. 1998). Subsequently VA opsin was shown to be expressed within the pineal organ and epithalamic/hypothalamic regions of the teleost brain (Philp *et al*. 2000*b*), sites strongly implicated as photoreceptive in fish. Similar findings were reported in the zebrafish (Kojima *et al*. 2000).

Two VA opsin isoforms were isolated in zebrafish, a long (VAL) and short (VAS) form, which vary in the length of their C-terminal tails (74 and seven amino acids, respectively). Both isoforms were functionally expressed in human embryonic kidney cells 293S but only VAL appeared capable of forming a photopigment when reconstituted with 11-*cis*-retinal (Kojima *et al*. 2000). Studies of several other teleosts, such as carp (*Cyprinus carpio*) (Moutsaki *et al*. 2000), smelt (*Plecoglossus altivelis*) (Minamoto & Shimizu 2002) and roach (*Rutilus rutilus*) (Jenkins *et al*. 2003) have confirmed the existence of different isoforms of VA opsins. In all cases, the shorter isoforms appear to be generated by intron retention at a splice site. A comparison of the known teleost sequences indicates that they fall into two groups, one consisting of zebrafish, roach and carp, the other of smelt and salmon. This split might be explained by the identification of a second VA gene in zebrafish, named VAL-opsin B by the authors (Kojima *et al*. 2008) (table 1). The newly isolated gene clades with the smelt and salmon sequences. The functional significance of this gene duplication in the teleost genome remains unclear.

These results in teleost fish prompted the search for orthologues of VA opsin in other vertebrate classes, but until recently attempts have met with failure. This restricted taxonomic distribution of the VA opsins was puzzling as most other opsins classes span multiple vertebrate taxa. Recent and unpublished studies have led to the isolation of the full-length sequence of chicken VA. The gene contains an open reading frame of 972 base pairs and encodes a predicted protein of 323 amino acids. Further studies have also identified VA-like genes in the Amphibia (*Xenopus tropicalis*), Reptilia (*Anolis carolinensis*) and the Elasmobranchii (*Callorhinchus milii*), but have failed to find any VA homologues within the mammalian lineage (S. Halford & R. G. Foster 2008, unpublished data). This surprising finding raises important questions as to the possible function of this opsin within vertebrate taxa.

### (e) Encephalopsin/panopsin (Opn3)

*Opn3* was originally termed encephalopsin, and reported to be an extra-ocular opsin with strong expression in mouse brain and testis, with lower levels in the heart, liver and kidney (Blackshaw & Snyder 1999). However, a subsequent study demonstrated that *Opn3* was in fact expressed in the retina and in all tissues examined; hence the name ‘panopsin’ was proposed (Halford *et al*. 2001). A more recent study on human *OPN3* confirms the wide tissue distribution and in multiple sites within the retina including the rods and cones, the outer plexiform, inner plexiform and ganglion cell layers of the retina (White *et al*. 2008). A comparison of Opn3 with the visual opsins shows a low amino acid identity of approximately 30 per cent (table 1). Opn3 contains a lysine, at residue 299, which is required for Schiff base linkage. This residue is equivalent to position 296 in bovine rod opsin but the counterion, usually a glutamate at position 113, is replaced by an aspartate. This does not preclude the formation of a photopigment as an aspartate residue is also present in the UV photopigment of *Xenopus*. The function of Opn3/OPN3 is further complicated by considerable alternate splicing of the human *Opn3* gene (Kasper *et al*. 2002).

### (f) TMT opsin

Isolated organs and cell lines from zebrafish have been shown to exhibit circadian oscillations in clock gene expression that can be entrained to light (Whitmore *et al*. 2000). These data provide strong evidence for the existence of a photopigment within these cells. TMT opsin was isolated in 2003 as part of a study to identify the photopigment or pigments in these peripheral tissues (Moutsaki *et al*. 2003). The full-length sequence of TMT opsin was isolated from *Fugu* and encodes a predicted protein of 402 amino acids, containing all of the essential features of an opsin photopigment including a lysine residue at position 296. Interestingly the Schiff base counterion, usually a glutamate, is substituted by a tyrosine in both *Fugu* and zebrafish TMT. TMT opsin shows 33–39% identity when compared to other vertebrate opsins (table 1). The gene was given its name because it is expressed in the liver, kidney and heart as well as eye and brain, and to date, has only been isolated from teleost fish. Phylogenetic analysis reveals that it clades with Opn3 (figure 3), which also exhibits a multiple pattern of tissue expression. The function of this gene family remains completely unresolved.

### (g) Melanopsin (Opn4)

Melanopsin was originally isolated from the photosensitive melanophores of *Xenopus* (Provencio *et al*. 1998). Subsequently, orthologs of melanopsin were isolated from mammals and shown to be expressed in a subset of RGCs (Provencio *et al*. 2000). In mammals, light information reaches the master circadian pacemaker, the suprachiasmatic nuclei (SCN), through a dedicated monosynaptic pathway that originates in the retina and is called the RHT. The anatomy and distribution of these melanopsin expressing cells was very similar to the RGCs that form the RHT. Subsequent analysis showed that these RGCs, which only account for approximately 1 per cent of the total, are directly photosensitive (pRGCs) (Hattar *et al*. 2002). See below for further discussion. Mouse *Opn4* encodes a predicted protein of 521 amino acids and contains the lysine residue at position 337 necessary for Schiff base formation. Melanopsin has now been isolated from a range of species including zebrafish (Bellingham *et al*. 2002) and the chicken (Chaurasia *et al*. 2005). In all cases the Opn4 opsins contain a tyrosine at the counterion position, like peropsin and TMT opsin, rather than a glutamate. The melanopsins show a relatively low level of identity with the photopigment opsins, approximately 27 per cent (table 1). Recently two melanopsin genes, *Opn4m* and *Opn4x*, have been described in non-mammalian vertebrates (Bellingham *et al*. 2006). But to date only one form, *Opn4m*, has been isolated from the placental and marsupial mammals (Pires *et al*. 2007). Preliminary *in silico* analysis also suggests that the monotreme the platypus lacks the *Opn4x* gene (S. Halford & R.G. Foster unpublished data).

### (h) Neuropsin (Opn5)

Neuropsin (*Opn5*) was identified in 2003 using a bioinformatic approach (Tarttelin *et al*. 2003). *Opn5* encodes a predicted protein of 377 amino acids in the mouse and 354 amino acids in human, with the mouse having a longer C-terminal tail. All of the expected features of an opsin are conserved, but Opn5 shows only 25–30% identity to the vertebrate members of the opsin superfamily (table 1). RT-PCR suggests that *Opn5* is expressed in mouse testis, brain and eye and in human retina and brain. Further work is necessary to establish both the type of retinal cells that express *Opn5/OPN5* and whether it can function as a photopigment.

## 4. A novel retinal photoreceptor

The identification of multiple photoreceptors, and the non-rod, non-cone opsin-based photopigments across the vertebrates were not predicted. Indeed, the assumption until the early 1990s was that some form of conventional rod- or cone-like opsin would mediate all forms of vertebrate photoreception, both visual and extraocular. But perhaps the least expected result to emerge has been the discovery of another class of photoreceptor within the eye, quite distinct from the rods and cones. Until recently it was inconceivable to most vision biologists that there could be an unrecognized class of photoreceptor within the vertebrate eye. After all, the eye was the best understood part of the central nervous system. One hundred and fifty years of research had explained how we see: photons are detected by the rods and cones and their graded potentials are assembled into an image by inner retinal neurons, followed by advanced visual processing in the brain. But image detection is very different from the demands of irradiance detection. Rods and cones are highly sensitive radiance detectors, which rapidly adapt and can only integrate signals of a short duration. By contrast, the circadian system is relatively insensitive to light, requiring high intensity and long duration stimuli to bring about photoentrainment. The appreciation that the mammalian eye has to perform two quite radically different sensory tasks triggered a line of enquiry that ultimately led to the discovery of a population of pRGC which use the photopigment melanopsin. The key findings that have led to this discovery are outlined next.

### (a) Retinal mutant studies

Initial studies to determine which retinal photoreceptors mediate circadian photoentrainment took advantage of a naturally occurring mutation in mice, termed retinal degeneration (*rd/rd*). These animals lack rods, and show a greatly reduced number of cones. As might be expected, *rd/rd* mice fail to show any classical visual responses to light. Studies were then undertaken to determine whether the circadian system of *rd/rd* mice was similarly impaired. When a mouse is housed under a light–dark cycle, its circadian wheel running behaviour is entrained. As would be expected of a nocturnal species, mice are largely active during the dark and inactive in the light. Under conditions of constant darkness, entrainment is lost and circadian behaviour drifts or freeruns with a recurring period approximately 23.5 h, starting its activity cycle approximately 0.5 h earlier every day. If the mouse is then exposed to a pulse of light shortly after activity onset it will delay the onset of activity the following day. The magnitude of this phase delay (Δ*ϕ*) is intensity dependent and can be used to determine the sensitivity of circadian responses to light. Both the wavelength and intensity of the light pulse can be systematically varied and the effect of these treatments on delaying wheel running behaviour can be assessed in a dose-dependent manner to produce a series of irradiance response curves.

Initial studies on photoentrainment in *rd/rd* mice (C3H) and wild-type controls (C57) demonstrated that these mice could still entrain, although the threshold for entrainment in *rd/rd* animals was approximately 2 log units higher (Ebihara & Tsuji 1980). The difference in the genetic background in these animals (C3H versus C57) appears to account for this difference in sensitivity (Foster & Helfrich-Forster 2001). Remarkably, in mice of the same genetic background, the massive loss of classical photoreceptors in the *rd/rd* mutants had little or no effect on the ability of the mice to either entrain to a light–dark cycle or phase-shift circadian rhythms in wheel running behaviour (Foster *et al*. 1991). Their irradiance response curves were indistinguishable from congenic wild-type controls, while eye loss completely blocked all effects of light on the clock (Foster *et al*. 1991). These studies demonstrated that the processing of light for circadian and visual responses must be different and hinted at the fact that there may be another class of ocular photoreceptor. Such suggestions were met with considerable scepticism and the favoured explanation was that as approximately 5 per cent of the cones survive in the retina of *rd/rd* mice (Carter-Dawson *et al*. 1978), it is probable that only a small number of photoreceptors are necessary for photoentrainment. Action spectrum studies on the spectral sensitivity of phase-shifting responses in *rd/rd* mice were subsequently conducted, suggesting a maximum sensitivity at either 511 or 480 nm, but these studies again failed to exclude the possibility of a residual cone contribution to these responses (Provencio & Foster 1995; Yoshimura & Ebihara 1996). The development of a mouse model lacking all rods and cones, the *rd/rd cl*, finally resolved these issues, and demonstrated that both phase-shifting responses and pineal melatonin suppression in response to light were apparently normal even when rod or cone opsins were undetectable (Freedman *et al*. 1999; Lucas *et al*. 1999).

The results from *rd/rd cl* mice provided the conclusive evidence that an additional photoreceptor exists within the mammalian eye and the conceptual framework for a host of further studies, including the finding that non-rod, non-cone photoreceptors do more than regulate the circadian system. Two examples are listed here: (i) In mammals, light-induced pupil constriction is regulated by both rods and cones, but still occurs in animals showing profound damage to these photoreceptors. Not unreasonably, it was assumed that the residual pupil light response was owing to the survival of a few rod and/or cone photoreceptors. Pupil measurements were undertaken in *rd/rd cl* mice and showed that these animals maintained a pupillary light response. Although less sensitive than congenic wild-type animals, *rd/rd cl* mice retained the ability to fully constrict their pupils (Lucas *et al*. 2001). (ii) Nocturnal rodents will inhibit their general activity when exposed to light during the night. This response, called masking, is thought to complement circadian entrainment by ensuring that activity is restricted to the hours of darkness or near darkness. Masking may be particularly important in environments where day length changes rapidly, and circadian behaviour may have difficulty keeping up with the expanding photoperiod (Mrosovsky 1999). Masking experiments were undertaken in *rd/rd cl* mice and demonstrated that there is marked inhibition of activity upon exposure to light presented two hours after normal lights off (Mrosovsky *et al*. 2001). Thus phase shifting, melatonin suppression, pupil constriction, masking and a number of other responses to light, such as sleep regulation (Lupi *et al*. 2008) are either intact or retained at some degree in mice lacking all their rods and cones.

The first action spectrum to be published on *rd/rd cl* mice was for pupil constriction, and the results described an opsin/vitamin A-based photopigment with a *λ*_max_ in the blue part of the spectrum near 480 nm (opsin photopigment/OP^480^). The known visual pigments of the mouse have *λ*_max_ values of 360, 508 and 498 nm for the ultraviolet-sensitive cone, long-wavelength sensitive cone and rod pigments, respectively. None of these classical photoreceptors could account for the pupillary responses to light (Lucas *et al*. 2001). Since 2001, a plethora of action spectra from mice to man have been deduced for a range of irradiance responses to light. These include the light responses of pRGCs in mice (Hattar *et al*. 2003), rats (Berson *et al*. 2002) and primates (Dacey *et al*. 2005) spanning pupil constriction, phase shifting circadian rhythms, plasma melatonin suppression, together with irradiance dependent regulation of human retinal cone function (Hankins & Lucas 2002). All these action spectra point to the existence of a single novel opsin photopigment with a *λ*_max_ of around 480 nm. A single invariant spectral sensitivity for the pRGCs is in marked contrast to the cone pigments, which are highly divergent and appear spectrally tuned in a species-specific manner. It remains unclear what ecological advantage this wavelength might confer on such diverse species. One possibility is that the pRGCs are tuned to the dominant wavelength of light at twilight. When the sun is close to the horizon there is relative enrichment of ‘blue’ light in the dome of the sky because of the preferential scattering of short wavelengths of light passing obliquely through the atmosphere.

### (b) Photosensitive retinal ganglion cells

The identification of the cells mediating non-rod, non-cone responses to light was provided by two sets of experiments. Studies on the rat used retrograde tracers injected into the SCN coupled with single cell recordings on isolated retina in which rod and cone responses were pharmacologically blocked (Berson *et al*. 2002). Parallel studies were undertaken using calcium imaging on the *rd/rd cl* retina (Sekaran *et al*. 2003). Both approaches identified a population of RGCs which responded directly to light. Significantly these pRGCs expressed the photopigment melanopsin (Hattar *et al*. 2002).

### (c) Melanopsin knockout studies

The essential data that melanopsin plays a critical role in the transduction of light information in pRGCs came from gene ablation studies. Melanopsin knockout mice (*Opn4^−/−^*) exhibited attenuated phase-shifting and pupillary responses to light, as well as reduced period lengthening in constant light (LL) (Panda *et al*. 2002; Ruby *et al*. 2002; Lucas *et al*. 2003). However, the critical involvement of melanopsin in photoreception came from triple-knockout studies, lacking rods, cones and melanopsin. These animals were totally unresponsive to light, demonstrating that melanopsin is in some way essential for pRGC photosensitivity (Hattar *et al*. 2003), but precisely what function melanopsin was playing was only finally resolved by using functional expression studies.

### (d) Melanopsin expression studies

The first investigation of the biochemistry of melanopsin involved expression of melanopsin in COS cells and reconstitution with 11-*cis*-retinal, an approach which has been particularly successful with visual pigments. This study produced a functional photopigment that was capable of activating transducin with a *λ*_max_ between 420 and 440 nm, an absorption maxima considerably shifted away from OP^480^ (Newman *et al*. 2003). The discrepancy in *λ*_max_ between spectroscopy and action spectra, coupled with low pigment yields, prompted other researchers to investigate whether expression of melanopsin alone was enough to confer photosensitivity. Quite independently, three groups combined the expression of melanopsin protein with physiological assays of cellular photosensitivity. All three studies showed that melanopsin transfection can confer photosensitivity to non-photosensitive cell types (Neuro-2a; HEK293-TRPC3; *Xenopus* oocyte) (Melyan *et al*. 2005; Panda *et al*. 2005; Qiu *et al*. 2005). In addition, these groups were able to show that specific forms of retinal (especially 11-*cis*-retinal) are needed for these responses to light, that light will ultimately trigger the release of intracellular calcium, and that this may involve a G_q_-type G-protein rather than transducin. Furthermore, melanopsin acts as a bistable pigment able to regenerate (recycle) its chromophore (11-*cis*-retinal) using all-*trans*-retinal and long-wavelength light in a manner reminiscent of the invertebrate photopigments (Melyan *et al*. 2005). In this regard melanopsin may be unique among mammalian photopigments in forming a stable association with all-*trans*-retinal.

Expression studies on human melanopsin suggest that the *λ*_max_ of light responses is close to 420–430 nm, and in this regard the findings were similar to those obtained by Newman and colleagues (Melyan *et al*. 2005). The studies on murine melanopsin, however, showed an action spectrum for light responses that exhibited a *λ*_max_ very close to 480 nm (Qiu *et al*. 2005). The current consensus from the various groups is that something about the local environment in which melanopsin is reconstituted is important in determining its *λ*_max_.

### (e) Melanopsin phototransduction

Most recently, research has turned to the phototransduction cascade used by melanopsin. Rod and cone opsins mediate a phototransduction cascade that involves the activation of transducin (a member of the G_i_/G_0_ class of G-proteins), phosphodiesterase and closure of cyclic nucleotide gated channels and a hyperpolarizing membrane potential. By contrast, invertebrate phototransduction, most extensively characterized in *Drosophila*, involves activation of a G_q_/G_11_-type G-protein, activation of phospholipase C (PLC), gating of transient receptor potential (TRP) channels and the depolarization of membrane potential (Hardie & Raghu 2001). Interestingly, the melanopsins appear to share some of the key characteristics of an invertebrate-like signal transduction pathway. Both pRGCs and cells transfected with melanopsin show depolarizing responses to light and, as discussed in §4*d*, melanopsin displays chromophore bistability, another feature of the invertebrate photopigments. Largely by analogy, it was proposed that melanopsin could be coupled to a G-protein of the G_q_/G_11_ class (for review see Peirson & Foster 2006). While not conclusive, there is support for this from the expression studies. For example, melanopsin responses are greatly attenuated (although not blocked) by antibodies against G_q_/G_11_ G-proteins (but not by antibodies to G_i_/G_0_) (Panda *et al*. 2003). In Neuro-2a cells, the use of G_i_/G_0_ blockers fails to inhibit melanopsin-dependent light responses (Melyan *et al*. 2005), while G_q_/G_11_ agonists fully blocked the melanopsin-dependent light responses in HEK293-TRPC3 cells (Qiu *et al*. 2005). Collectively these initial results suggest that the G_q_/G_11_ G-proteins could be activated by melanopsin-dependent phototransduction. It is important to stress, however, that the coupling potential in non-native host environments might not reflect the native pRGCs. Downstream of the G-protein, melanopsin-dependent light responses are greatly attenuated or blocked in *Xenopus* oocytes and HEK293-TRPC3 cells by PLC inhibitors (Panda *et al*. 2005; Qiu *et al*. 2005). Furthermore, co-expression of melanopsin with TRPC3 in *Xenopus* oocytes (similar to the *Drosophila* TRP channels) shows that TRPC3 channels can generate a light-activated photocurrent in the presence of melanopsin (Panda *et al*. 2005; Qiu *et al*. 2005). Collectively, a partial model of the phototransduction cascade has emerged, suggesting that light activated melanopsin may interact with G_q_/G_11_ that in turn activates a PLC-β. PLC-β generates inositol triphosphate (IP_3_) and diacylglycerol (DAG), which may ultimately modulate a TRPC channel, possibly via a protein kinase C (PKC). Most recently combined pharmacolgical and anatomical approaches have suggested TRPC7 as the channel (Sekaran *et al*. 2007). In addition, a microarray-based approach has been used to investigate the transcriptional realignment that occurs in the *rd/rd cl* mouse eye following a light pulse. This approach identified a number of candidate genes/proteins that might be associated with the melanopsin cascade. Among these was the atypical protein kinase C zeta (*Prkcz*). Remarkably the genetic ablation of *Prkcz* mimics precisely the melanopsin knock-out phenotype in a battery of behavioural and pupillometric tests (Peirson *et al*. 2007). Why an ‘invertebrate-like’ signalling pathway, rather than a more conventional vertebrate phototransduction pathway, is employed by the pRGCs remains an intriguing sensory question and may be relevant to understanding the evolutionary origins of the melanopsin/pRGCs photoreceptor system (Arendt 2003).

## 5. Evolutionary considerations of non-image-forming photoreception

Given the multiplicity of photoreceptive tissues in the non-mammalian vertebrates, why have these been lost in the mammalian lineage? One possible explanation may be related to the early evolutionary history of the mammals and their passage through a ‘nocturnal bottleneck’. Modern mammals seem to have been derived from nocturnal insectivorous or omnivorous animals about 100 million years ago (Young 1962). Pineal and deep-brain photoreceptors would have been perfectly adequate for monitoring changes in diurnal light conditions but may not have been sufficiently sensitive to discriminate twilight changes in mammals living in burrows or otherwise concealed during the day. The occupation of the nocturnal realm may have led to the loss of the extraocular photoreceptors and the exclusive reliance on irradiance detection by the pRGCs (Foster & Menaker 1993; Menaker *et al*. 1997). But of course this explanation does not explain why the vertebrates evolved so many photosensitive tissues in the first place. In this context it is worth emphasizing that the sensory task of reliable irradiance detection is not trivial, and extracting time-of-day information from environmental irradiance is even more complex. For example, during twilight, the quality of light changes in three important respects: (i) the amount of light; (ii) the spectral composition of light; (iii) and the source of light (i.e. the position of the sun). These parameters all change in a systematic way and could be used by the circadian system to detect the phase of twilight and hence time of day (Roenneberg & Foster 1997). However, each of these parameters is subject to considerable sensory ‘noise’. The sources of this noise are summarized in table 2. Clearly, the impact of this noise will depend upon the organism and the environment it inhabits. Integrating the information from a multiplicity of photoreceptors, which collect light from different regions of the environment, with differing integration times, and tuned to different spectral channels will act to reduce signal noise and hence provide a more reliable measure of environmental irradiance. The non-mammalian vertebrates might integrate light information from the pineal, deep brain and eyes for reliable time-of-day detection, and there is good evidence for this in birds (Menaker & Underwood 1976). In mammals, twilight detection is either less precise, because of the reliance on a single photoreceptor type, or the pRGCs themselves show heterogeneity in their responses to light, for which there is also good evidence (Sekaran *et al*. 2005).

**Table 2.** The major sources of noise associated with the detection of environmental irradiance. The main sources of signal noise for irradiance detection are listed with examples. In each case the impact of this noise will depend upon the organism, its developmental state and the environment that it inhabits. Integrating the information from multiple photoreceptors, which collect light from different regions of the environment, having differing integration times, and tuned to different spectral channels will act to reduce signal noise.

source of signal noise	examples
fluctuation in the light signal	cloud cover, day-length
extraneous light signals	starlight, moonlight, lightning
receptor noise	variation in external temperature
sensory adaptation	receptor habituation
behavioural noise	emergence from burrow, place of rest, feeding etc.

Opsins in general have evolved to mediate specific photoreceptive tasks in different light environments (Lythgoe 1979). For example, in environments where the spectral composition of the light is restricted, such as in deep water, the *λ*_max_ of photopigments is spectrally tuned to match the maximum available photon flux around 480 nm (Douglas & Partridge 1997; Hope *et al*. 1997; Hunt *et al*. 2001). Whether similar spectral tuning arguments can be used to understand the *λ*_max_ of the non-image-forming photopigments remains an intriguing question. Many photoreceptors involved in non-image-forming tasks appear to peak close to 480 nm, with a spread ranging from 460 to 530 nm (Shand & Foster 1999). In pineal and deep-brain photoreceptors the light available will be dominated by the transmission of the overlying tissues. This is primarily influenced by two factors. Firstly, short wavelength light is scattered more than longer wavelength light, resulting in relatively more light of long wavelengths penetrating to reach intracranial photoreceptors. Secondly, light will be modified by light-absorbing pigments before reaching these photoreceptors. The most important such pigment is haemoglobin. Haemoglobin has a transmission window between 460 and 540 nm, peaking around 490 nm (Hartwig & van Veen 1979; Foster & Follett 1985). This transmission window may have exerted a strong selection pressure on the spectral tuning of deep brain and pineal photoreceptors. But this cannot be the entire explanation as many non-image-forming photoreceptors are directly exposed to environmental light, such as the pRGCs in the eye or dermal photoreceptors, and these have *λ*_max_ around 480 nm.

Changes in the amount and spectral composition of environmental irradiance occur throughout the diurnal cycle. As well as the obvious gross changes in irradiance at twilight (approx. 6 log units), changes in the spectral composition of light also occur and are known as the Chappuis effect (Lythgoe 1979). As the sun's rays must pass through a thicker layer of the atmosphere when the sun is lower in the sky, the absorption of light by ozone (500–650 nm) results in a relative enrichment of shorter wavelength light (<500 nm) at twilight (Munz & McFarland 1977). As changes in the light environment at twilight are critical for photoentrainment (Roenneberg & Foster 1997), ‘twilight detectors’ spectrally tuned to the blue part of the spectrum could allow increased photon capture and hence an increase in signal-to-noise detection. Perhaps, however, it is not simply the amount of light that is being detected at twilight but rather its change in spectral quality. Evidence for spectral discrimination, a chromatic response, was first shown in the pineal organ of fish (Meissl & Yanez 1994) and more recently in the parietal eye of lizards (Su *et al*. 2006). These chromatic responses could arise from an interaction between different photopigments with differing *λ*_max_ or a single bistable photopigment. Significantly, melanopsin appears to act as a bi-stable pigment, able to regenerate its chromophore using all-*trans*-retinal and long-wavelength light (Melyan *et al*. 2005). This photoreversal capacity of melanopsin has also been observed with spectroscopic approaches in the case of *Amphioxus* melanopsin (Koyanagi *et al*. 2004). If the two stable states of melanopsin are capable of interacting with different downstream signalling transduction pathways, this may provide an alternative means of attaining spectral discrimination.

The spectral tuning of vertebrate opsins will also be influenced by their evolutionary history (Goldsmith 1990). For example, key amino acid residues influencing spectral tuning sites may provide structural or functional properties, such that any mutation of these residues will be deleterious to protein function. Additionally, there will be trade-offs between structure and function that will influence spectral tuning. Scotopic vision is limited by dark noise produced by spontaneous thermal isomerizations of the retinal chromophore (Barlow *et al*. 1993). Long wavelength sensitive photopigments have been suggested to be more prone to dark noise owing to their lower excitation energy (Barlow 1957). Thus the spectral tuning of the non-visual opsins, like the visual opsins, will always be a compromise between functional constraints and the photon flux of the light environment (Lythgoe 1984; Goldsmith 1990; Barlow *et al*. 1993).

## 6. Conclusions

Considerable progress has been made in the last decade in characterizing the photoreceptors and photopigments mediating non-image-forming responses to light, such as photoentrainment. While over a dozen different opsin photopigments have been identified in recent years (table 1), we are only just beginning to understand what roles these proteins play in the signalling of light information. Perhaps the greatest single advance has been the identification of a third photoreceptive system in the vertebrate eye, the melanopsin-expressing pRGCs, which mediate a range of irradiance detection tasks ranging from photoentrainment, pineal melatonin suppression, pupil constriction and the modulation of arousal states and sleep induction (Altimus *et al*. 2008; Lupi *et al*. 2008). By contrast, the photopigments responsible for non-image-forming responses to light in many non-mammalian species remain poorly characterized. Molecular studies are required to determine whether these opsins can form photopigments or whether they act as photoisomerases or retinal carrier proteins (Foster & Bellingham 2002). However a functional analysis of these opsins requires more than biochemistry. If we are to place these remarkable photoreceptors into any sort of evolutionary context we will need a much better understanding of their sensory ecology. We now appreciate that these photoreceptors do more than act as simple photon counters—but beyond this—any detailed understanding is lacking.

## References

[RSTB20090050C1] Altimus C. M. Guler A. D. Villa K. L. McNeill D. S. Legates T. A. Hattar S. 2008 Rods–cones and melanopsin detect light and dark to modulate sleep independent of image formation. Proc. Natl Acad. Sci. USA 105, 19 998–20 003 (doi:10.1073/pnas.0808312105) 10.1073/pnas.0808312105 PMC2596746 19060203

[RSTB20090050C2] Arendt J. 1998 Melatonin and the pineal gland: influence on mammalian seasonal and circadian physiology. Rev. Reprod. 3, 13–22 (doi:10.1530/ror.0.0030013) 9509985 10.1530/ror.0.0030013

[RSTB20090050C3] Arendt D. 2003 Evolution of eyes and photoreceptor cell types. Int. J. Dev. Biol. 47, 563–571 14756332

[RSTB20090050C4] Aschoff J. 1984 Circadian timing. Ann. N. Y. Acad. Sci. 423, 442–468 (doi:10.1111/j.1749-6632.1984.tb23452.x) 6588808 10.1111/j.1749-6632.1984.tb23452.x

[RSTB20090050C5] Barlow H. B. 1957 Purkinje shift and retinal noise. Nature 179, 255–256 (doi:10.1038/179255b0) 13407693 10.1038/179255b0

[RSTB20090050C6] Barlow R. B. Birge R. R. Kaplan E. Tallent J. R. 1993 On the molecular origin of photoreceptor noise. Nature 366, 64–66 (doi:10.1038/366064a0) 8232538 10.1038/366064a0

[RSTB20090050C7] Barr L. Alpern M. 1963 Photosensitivity of the frog iris. J. Gen. Physiol. 46, 1249–1265 (doi:10.1085/jgp.46.6.1249) 14043001 10.1085/jgp.46.6.1249 PMC2195315

[RSTB20090050C8] Bellingham J. Foster R. G. 2002 Opsins and mammalian photoentrainment. Cell Tissue Res. 309, 57–71 (doi:10.1007/s00441-002-0573-4) 12111537 10.1007/s00441-002-0573-4

[RSTB20090050C9] Bellingham J. Whitmore D. Philp A. R. Wells D. J. Foster R. G. 2002 Zebrafish melanopsin: isolation, tissue localisation and phylogenetic position. Brain Res. Mol. Brain Res. 107, 128–136 (doi:10.1016/S0169-328X(02)00454-0) 12487121 10.1016/s0169-328x(02)00454-0

[RSTB20090050C10] Bellingham J. Wells D. J. Foster R. G. 2003 In silico characterisation and chromosomal localisation of human RRH (peropsin)—implications for opsin evolution. BMC Genomics 4, 3 (doi:10.1186/1471-2164-4-3) 12542842 10.1186/1471-2164-4-3 PMC149353

[RSTB20090050C11] Bellingham J. 2006 Evolution of melanopsin photoreceptors: discovery and characterization of a new melanopsin in nonmammalian vertebrates. PLoS Biol. 4, e254 (doi:10.1371/journal.pbio.0040254) 16856781 10.1371/journal.pbio.0040254 PMC1514791

[RSTB20090050C12] Benoit J. 1964 The role of the eyes and of the hypothalamus in the photostimulation of gonads in the duck. Ann. N. Y. Acad. Sci. 117, 204–215 (doi:10.1111/j.1749-6632.1964.tb48175.x) 14196641 10.1111/j.1749-6632.1964.tb48175.x

[RSTB20090050C13] Berson D. M. Dunn F. A. Takao M. 2002 Phototransduction by retinal ganglion cells that set the circadian clock. Science 295, 1070–1073 (doi:10.1126/science.1067262) 11834835 10.1126/science.1067262

[RSTB20090050C14] Bito L. Z. Turansky D. G. 1975 Photoactivation of pupillary constriction in the isolated in vitro iris of mammal (*Mesocricetus auratus*). Comp. Biochem. Physiol. A 50, 407–413 (doi:10.1016/0300-9629(75)90034-1) 234353 10.1016/0300-9629(75)90034-1

[RSTB20090050C15] Blackshaw S. Snyder S. H. 1997 Parapinopsin, a novel catfish opsin localized to the parapineal organ, defines a new gene family. J. Neurosci. 17, 8083–8092 9334384 10.1523/JNEUROSCI.17-21-08083.1997 PMC6573767

[RSTB20090050C16] Blackshaw S. Snyder S. H. 1999 Encephalopsin: a novel mammalian extraretinal opsin discretely localized in the brain. J Neurosci. 19, 3681–3690 10234000 10.1523/JNEUROSCI.19-10-03681.1999 PMC6782724

[RSTB20090050C17] Bowmaker J. Hunt D. M. 1999 Molecular biology of photoreceptor spectral sensitivity. In Adaptive mechanisms in the ecology of vision (eds Archer S. N. Djamgoz M. B. A. Loew E. R. Partridge J. C. Vallerga S.), pp. 439–462 Dordrecht, The Netherlands: Kluwer Academic Publishers

[RSTB20090050C18] Carter-Dawson L. D. LaVail M. M. Sidman R. L. 1978 Differential effect of the rd mutation on rods and cones in the mouse retina. Invest Ophthalmol. Vis. Sci. 17, 489–498 659071

[RSTB20090050C19] Chaurasia S. S. 2005 Molecular cloning, localization and circadian expression of chicken melanopsin (*Opn4*): differential regulation of expression in pineal and retinal cell types. J. Neurochem. 92, 158–170 (doi:10.1111/j.1471-4159.2004.02874.x) 15606905 10.1111/j.1471-4159.2004.02874.x

[RSTB20090050C20] Dacey D. M. Liao H. W. Peterson B. B. Robinson F. R. Smith V. C. Pokorny J. Yau K. W. Gamlin P. D. 2005 Melanopsin-expressing ganglion cells in primate retina signal colour and irradiance and project to the LGN. Nature 433, 749–754 (doi:10.1038/nature03387) 15716953 10.1038/nature03387

[RSTB20090050C21] Dartnall H. 1953 The interpretation of spectral sensitivity curves. Br. Med. Bull. 9, 24–30 13032421 10.1093/oxfordjournals.bmb.a074302

[RSTB20090050C22] Dodt E. Meissl H. 1982 The pineal and parietal organs of lower vertebrates. Experientia 38, 996–1000 (doi:10.1007/BF01955342) 6751863 10.1007/BF01955342

[RSTB20090050C23] Douglas R. H. Partridge J. C. 1997 On the visual pigments of deep-sea fish. J. Fish Biol. 50, 68–85 (doi:10.1111/j.1095-8649.1997.tb01340.x)

[RSTB20090050C24] Ebihara S. Tsuji K. 1980 Entrainment of the circadian activity rhythm to the light cycle: effective light intensity for a Zeitgeber in the retinal degenerate C3H mouse and the normal C57BL mouse. Physiol. Behav. 24, 523–527 (doi:10.1016/0031-9384(80)90246-2) 7375573 10.1016/0031-9384(80)90246-2

[RSTB20090050C25] Foster R. Bellingham J. 2002 Opsins and melanopsins. Curr. Biol. 12, R543–R544 (doi:10.1016/S0960-9822(02)01047-3) 12194831 10.1016/s0960-9822(02)01047-3

[RSTB20090050C26] Foster R. G. Follett B. K. 1985 The involvement of a rhodopsin-like photopigment in the photoperiodic response of the Japanese quail. J. Comp. Physiol 157A, 519–528 (doi:10.1007/BF00615153)

[RSTB20090050C27] Foster R. G. Helfrich-Forster C. 2001 The regulation of circadian clocks by light in fruitflies and mice. Phil. Trans. R. Soc. B 356, 1779–1789 (doi:10.1098/rstb.2001.0962) 11710985 10.1098/rstb.2001.0962 PMC1088554

[RSTB20090050C28] Foster R. G. Menaker M. 1993 Circadian photoreception in mammals and other vertebrates. In Light and biological rhythms in man (ed. Wetterberg L.), pp. 73–91 Oxford, UK and New York: Pergamon

[RSTB20090050C29] Foster R. G. Timmers A. M. Schalken J. J. De Grip W. J. 1989 A comparison of some photoreceptor characteristics in the pineal and retina. II. The Djungarian hamster (*Phodopus sungorus*). J. Comp. Physiol 165A, 565–572 (doi:10.1007/BF00611242) 2527978 10.1007/BF00611242

[RSTB20090050C30] Foster R. G. Provencio I. Hudson D. Fiske S. De Grip W. Menaker M. 1991 Circadian photoreception in the retinally degenerate mouse (rd/rd). J. Comp. Physiol 169A, 39–50 (doi:10.1007/BF00198171) 1941717 10.1007/BF00198171

[RSTB20090050C31] Foster R. G. Provencio I. Bovee-Geurts P. H. DeGrip W. J. 2003 The photoreceptive capacity of the developing pineal gland and eye of the golden hamster (*Mesocricetus auratus*). J. Neuroendocrinol. 15, 355–363 (doi:10.1046/j.1365-2826.2003.01004.x) 12622834 10.1046/j.1365-2826.2003.01004.x

[RSTB20090050C32] Freedman M. S. Lucas R. J. Soni B. von Schantz M. Munoz M. David-Gray Z. Foster R. 1999 Regulation of mammalian circadian behavior by non-rod, non-cone, ocular photoreceptors. Science 284, 502–504 (doi:10.1126/science.284.5413.502) 10205061 10.1126/science.284.5413.502

[RSTB20090050C33] Frigato E. Vallone D. Bertolucci C. Foulkes N. S. 2006 Isolation and characterization of melanopsin and pinopsin expression within photoreceptive sites of reptiles. Naturwissenschaften 93, 379–385 (doi:10.1007/s00114-006-0119-9) 16688437 10.1007/s00114-006-0119-9

[RSTB20090050C34] Goldsmith T. H. 1990 Optimization, constraint, and history in the evolution of eyes. Q. Rev. Biol. 65, 281–322 (doi:10.1086/416840) 2146698 10.1086/416840

[RSTB20090050C35] Halford S. Freedman M. Bellingham J. Inglis S. Poopalasundaram S. Soni B. Foster R. Hunt D. 2001 Characterization of a novel human opsin gene with wide tissue expression and identification of embedded and flanking genes on chromosome 1q43. Genomics 72, 203–208 (doi:10.1006/geno.2001.6469) 11401433 10.1006/geno.2001.6469

[RSTB20090050C36] Hankins M. W. Lucas R. J. 2002 The primary visual pathway in humans is regulated according to long-term light exposure through the action of a nonclassical photopigment. Curr. Biol. 12, 191–198 (doi:10.1016/S0960-9822(02)00659-0) 11839270 10.1016/s0960-9822(02)00659-0

[RSTB20090050C37] Hao W. Fong H. K. 1996 Blue and ultraviolet light-absorbing opsin from the retinal pigment epithelium. Biochemistry 35, 6251–6256 (doi:10.1021/bi952420k) 8639565 10.1021/bi952420k

[RSTB20090050C38] Hao W. Fong H. K. 1999 The endogenous chromophore of retinal G protein-coupled receptor opsin from the pigment epithelium. J. Biol. Chem. 274, 6085–6090 (doi:10.1074/jbc.274.10.6085) 10037690 10.1074/jbc.274.10.6085

[RSTB20090050C39] Hardie R. C. Raghu P. 2001 Visual transduction in Drosophila. Nature 413, 186–193 (doi:10.1038/35093002) 11557987 10.1038/35093002

[RSTB20090050C40] Hargrave P. A. McDowell J. H. 1992 Rhodopsin and phototransduction: a model system for G protein-linked receptors. FASEB J. 6, 2323–2331 1544542 10.1096/fasebj.6.6.1544542

[RSTB20090050C41] Hartwig H.-G. van Veen T. 1979 Spectral characteristics of visible radiations penetrating into the brain and stimulating extra-retinal photoreceptors. J. Comp. Physiol. A 120, 277–282 (doi:10.1007/BF00614615)

[RSTB20090050C42] Hattar S. Liao H. W. Takao M. Berson D. M. Yau K. W. 2002 Melanopsin-containing retinal ganglion cells: architecture, projections, and intrinsic photosensitivity. Science 295, 1065–1070 (doi:10.1126/science.1069609) 11834834 10.1126/science.1069609 PMC2885915

[RSTB20090050C43] Hattar S. 2003 Melanopsin and rod–cone photoreceptive systems account for all major accessory visual functions in mice. Nature 424, 75–81 (doi:10.1038/nature01761) 10.1038/nature01761 PMC2885907 12808468

[RSTB20090050C44] Higgins D. G. Thompson J. D. Gibson T. J. 1996 Using CLUSTAL for multiple sequence alignments. Methods Enzymol. 266, 383–402 (doi:10.1016/S0076-6879(96)66024-8) 8743695 10.1016/s0076-6879(96)66024-8

[RSTB20090050C45] Hope A. J. Partridge J. C. Dulai K. S. Hunt D. M. 1997 Mechanisms of wavelength tuning in the rod opsins of deep-sea fishes. Proc. R. Soc. Lond. B 264, 155–163 (doi:10.1098/rspb.1997.0023) 10.1098/rspb.1997.0023 PMC1688238 9061967

[RSTB20090050C46] Hunt D. M. Dulai K. S. Partridge J. C. Cottrill P. Bowmaker J. K. 2001 The molecular basis for spectral tuning of rod visual pigments in deep-sea fish. J. Exp. Biol. 204, 3333–3344 11606607 10.1242/jeb.204.19.3333

[RSTB20090050C47] Isoldi M. C. Rollag M. D. Castrucci A. M. Provencio I. 2005 Rhabdomeric phototransduction initiated by the vertebrate photopigment melanopsin. Proc. Natl Acad. Sci. USA 102, 1217–1221 (doi:10.1073/pnas.0409252102) 15653769 10.1073/pnas.0409252102 PMC545850

[RSTB20090050C48] Jenkins A. Munoz M. Tarttelin E. E. Bellingham J. Foster R. G. Hankins M. W. 2003 VA opsin, melanopsin, and an inherent light response within retinal interneurons. Curr. Biol. 13, 1269–1278 (doi:10.1016/S0960-9822(03)00509-8) 12906786 10.1016/s0960-9822(03)00509-8

[RSTB20090050C49] Jiang M. Pandey S. Fong H. K. 1993 An opsin homologue in the retina and pigment epithelium. Invest. Ophthalmol. Vis. Sci. 34, 3669–3679 8258527

[RSTB20090050C50] Kasper G. 2002 Different structural organization of the encephalopsin gene in man and mouse. Gene 295, 27–32 (doi:10.1016/S0378-1119(02)00799-0) 12242008 10.1016/s0378-1119(02)00799-0

[RSTB20090050C51] Kawamura S. Yokoyama S. 1997 Expression of visual and nonvisual opsins in American chameleon. Vision Res. 37, 1867–1871 (doi:10.1016/S0042-6989(96)00309-4) 9274772 10.1016/s0042-6989(96)00309-4

[RSTB20090050C52] Knowles A. Dartnall H. 1977 In The photobiology of vision New York, NY: Academic Press

[RSTB20090050C53] Kojima D. Mano H. Fukada Y. 2000 Vertebrate ancient-long opsin: a green-sensitive photoreceptive molecule present in zebrafish deep brain and retinal horizontal cells. J. Neurosci. 20, 2845–2851 10751436 10.1523/JNEUROSCI.20-08-02845.2000 PMC6772192

[RSTB20090050C54] Kojima D. Torii M. Fukada Y. Dowling J. E. 2008 Differential expression of duplicated VAL-opsin genes in the developing zebrafish. J. Neurochem. 104, 1364–1371 (doi:10.1111/j.1471-4159.2007.05093.x) 18036148 10.1111/j.1471-4159.2007.05093.x PMC2702163

[RSTB20090050C55] Korf H.-W. Moller M. 1984 The innervation of the mammalian pineal gland with special reference to central pinealopetal projections. Pineal Res. Rev. 2, 41–86

[RSTB20090050C56] Korf H. W. Foster R. G. Ekstrom P. Schalken J. J. 1985a Opsin-like immunoreaction in the retinae and pineal organs of four mammalian species. Cell Tissue Res. 242, 645–648 (doi:10.1007/BF00225432) 2934135 10.1007/BF00225432

[RSTB20090050C57] Korf H. W. Moller M. Gery I. Zigler J. S. Klein D. C. 1985b Immunocytochemical demonstration of retinal S-antigen in the pineal organ of four mammalian species. Cell Tissue Res. 239, 81–85 (doi:10.1007/BF00214906) 3967288 10.1007/BF00214906

[RSTB20090050C58] Korf H. W. Schomerus C. Stehle J. H. 1998 The pineal organ, its hormone melatonin, and the photoneuroendocrine system. Adv. Anat. Embryol. Cell Biol. 146, 1–100 9670565 10.1007/978-3-642-58932-4

[RSTB20090050C59] Koyanagi M. Kawano E. Kinugawa Y. Oishi T. Shichida Y. Tamotsu S. Terakita A. 2004 Bistable UV pigment in the lamprey pineal. Proc. Natl Acad. Sci. USA 101, 6687–6691 (doi:10.1073/pnas.0400819101) 15096614 10.1073/pnas.0400819101 PMC404106

[RSTB20090050C60] Lau K. C. So K. F. Campbell G. Lieberman A. R. 1992 Pupillary constriction in response to light in rodents, which does not depend on central neural pathways. J. Neurol. Sci. 113, 70–79 (doi:10.1016/0022-510X(92)90267-O) 1469457 10.1016/0022-510x(92)90267-o

[RSTB20090050C61] Lucas R. J. Freedman M. S. Munoz M. Garcia-Fernandez J. M. Foster R. G. 1999 Regulation of the mammalian pineal by non-rod, non-cone, ocular photoreceptors. Science 284, 505–507 (doi:10.1126/science.284.5413.505) 10205062 10.1126/science.284.5413.505

[RSTB20090050C62] Lucas R. J. Douglas R. H. Foster R. G. 2001 Characterization of an ocular photopigment capable of driving pupillary constriction in mice. Nat. Neurosci. 4, 621–626 (doi:10.1038/88443) 11369943 10.1038/88443

[RSTB20090050C63] Lucas R. J. Hattar S. Takao M. Berson D. M. Foster R. G. Yau K. W. 2003 Diminished pupillary light reflex at high irradiances in melanopsin-knockout mice. Science 299, 245–247 (doi:10.1126/science.1077293) 12522249 10.1126/science.1077293

[RSTB20090050C64] Lupi D. Oster H. Thompson S. Foster R. G. 2008 The acute light-induction of sleep is mediated by OPN4-based photoreception. Nat. Neurosci. 11, 1068–1073 19160505 10.1038/nn.2179

[RSTB20090050C65] Lythgoe J. 1979 The ecology of vision Oxford, UK: Oxford University Press

[RSTB20090050C66] Lythgoe J. N. 1984 Visual pigments and environmental light. Vision Res. 24, 1539–1550 (doi:10.1016/S0042-6989(84)80003-6) 6398560 10.1016/s0042-6989(84)80003-6

[RSTB20090050C67] Mano H. Kojima D. Fukada Y. 1999 Exo-rhodopsin: a novel rhodopsin expressed in the zebrafish pineal gland. Brain Res. Mol. Brain Res. 73, 110–118 (doi:10.1016/S0169-328X(99)00242-9) 10581404 10.1016/s0169-328x(99)00242-9

[RSTB20090050C68] Max M. McKinnon P. J. Seidenman K. J. Barrett R. K. Applebury M. L. Takahashi J. S. Margolskee R. F. 1995 Pineal opsin: a nonvisual opsin expressed in chick pineal. Science 267, 1502–1506 (doi:10.1126/science.7878470) 7878470 10.1126/science.7878470

[RSTB20090050C69] Max M. Surya A. Takahashi J. S. Margolskee R. F. Knox B. E. 1998 Light-dependent activation of rod transducin by pineal opsin. J. Biol. Chem. 273, 26 820–26 826 (doi:10.1074/jbc.273.41.26820) 10.1074/jbc.273.41.26820 9756926

[RSTB20090050C70] Meissl H. 1997 Photic regulation of pineal function. Analogies between retinal and pineal photoreception. Biol. Cell 89, 549–554 (doi:10.1016/S0248-4900(98)80158-5) 9673006

[RSTB20090050C71] Meissl H. Yanez J. 1994 Pineal photosensitivity: a comparison with retinal photoreception. Acta Neurobiol. Exp. 54, 19–29 7801789

[RSTB20090050C72] Melyan Z. Tarttelin E. E. Bellingham J. Lucas R. J. Hankins M. W. 2005 Addition of human melanopsin renders mammalian cells photoresponsive. Nature 433, 741–745 (doi:10.1038/nature03344) 15674244 10.1038/nature03344

[RSTB20090050C73] Menaker M. Underwood H. 1976 Extraretinal photoreception in birds. Photochem. Photobiol. 23, 299–306 (doi:10.1111/j.1751-1097.1976.tb07251.x) 10.1111/j.1751-1097.1976.tb07251.x 1273098

[RSTB20090050C74] Menaker M. Moreira L. F. Tosini G. 1997 Evolution of circadian organization in vertebrates. Braz. J. Med. Biol. Res. 30, 305–313 (doi./10.1590/S0100-879X1997000300003) 9246228 10.1590/s0100-879x1997000300003

[RSTB20090050C75] Minamoto T. Shimizu I. 2002 A novel isoform of vertebrate ancient opsin in a smelt fish *Plecoglossus altivelis*. Biochem. Biophys. Res. Commun. 290, 280–286 (doi:10.1006/bbrc.2001.6186) 11779166 10.1006/bbrc.2001.6186

[RSTB20090050C76] Moutsaki P. Bellingham J. Soni B. G. David-Gray Z. K. Foster R. G. 2000 Sequence, genomic structure and tissue expression of carp (Cyprinus carpio L.) vertebrate ancient (VA) opsin. FEBS Lett. 473, 316–322 (doi:10.1016/S0014-5793(00)01550-7) 10818232 10.1016/s0014-5793(00)01550-7

[RSTB20090050C77] Moutsaki P. Whitmore D. Bellingham J. Sakamoto K. David-Gray Z. K. Foster R. G. 2003 Teleost multiple tissue (tmt) opsin: a candidate photopigment regulating the peripheral clocks of zebrafish? Brain Res. Mol. Brain Res. 112, 135–145 (doi:10.1016/S0169-328X(03)00059-7) 12670711 10.1016/s0169-328x(03)00059-7

[RSTB20090050C78] Mrosovsky N. 1999 Masking: history, definitions, and measurement. Chronobiol. Int. 16, 415–429 (doi:10.3109/07420529908998717) 10442236 10.3109/07420529908998717

[RSTB20090050C79] Mrosovsky N. Lucas R. Foster R. 2001 Persistence of masking responses to light in mice lacking rods and cones. J. Biol. Rhythms 16, 585–587 (doi:10.1177/074873001129002277) 11760016 10.1177/074873001129002277

[RSTB20090050C80] Munz F. W. McFarland W. N. 1977 Evolutionary adaptations of fishes to the photic environment. In Handbook of sensory physiology (ed. Crescitelli F.), pp. 193–274 Berlin, Germany: Springer

[RSTB20090050C81] Nakamura A. Kojima D. Imai H. Terakita A. Okano T. Shichida Y. Fukada Y. 1999 Chimeric nature of pinopsin between rod and cone visual pigments. Biochemistry 38, 14 738–14 745 (doi:10.1021/bi9913496) 10.1021/bi9913496 10555955

[RSTB20090050C82] Nathans J. Hogness D. S. 1983 Isolation, sequence analysis, and intron–exon arrangement of the gene encoding bovine rhodopsin. Cell 34, 807–814 (doi:10.1016/0092-8674(83)90537-8) 6194890 10.1016/0092-8674(83)90537-8

[RSTB20090050C83] Nelson R. J. Zucker I. 1981 Absence of extra-ocular photoreception in diurnal and nocturnal rodents exposed to direct sunlight. Comp. Biochem. Physiol. 69A, 145–148 (doi:10.1016/0300-9629(81)90651-4)

[RSTB20090050C84] Newman L. A. Walker M. T. Brown R. L. Cronin T. W. Robinson P. R. 2003 Melanopsin forms a functional short-wavelength photopigment. Biochemistry 42, 12 734–12 738 (doi:10.1021/bi035418z) 10.1021/bi035418z 14596587

[RSTB20090050C85] Okada T. Ernst O. P. Palczewski K. Hofmann K. P. 2001 Activation of rhodopsin: new insights from structural and biochemical studies. Trends Biochem. Sci. 26, 318–324 (doi:10.1016/S0968-0004(01)01799-6) 11343925 10.1016/s0968-0004(01)01799-6

[RSTB20090050C86] Okano T. Yoshizawa T. Fukada Y. 1994 Pinopsin is a chicken pineal photoreceptive molecule. Nature 372, 94–97 (doi:10.1038/372094a0) 7969427 10.1038/372094a0

[RSTB20090050C87] Palczewski K. 2000 Crystal structure of rhodopsin: a G protein-coupled receptor. Science 289, 739–745 (doi:10.1126/science.289.5480.739) 10926528 10.1126/science.289.5480.739

[RSTB20090050C88] Panda S. Sato T. K. Castrucci A. M. Rollag M. D. DeGrip W. J. Hogenesch J. B. Provencio I. Kay S. A. 2002 Melanopsin (*Opn4*) requirement for normal light-induced circadian phase shifting. Science 298, 2213–2216 (doi:10.1126/science.1076848) 12481141 10.1126/science.1076848

[RSTB20090050C89] Panda S. 2003 Melanopsin is required for non-image-forming photic responses in blind mice. Science 301, 525–527 (doi:10.1126/science.1086179) 12829787 10.1126/science.1086179

[RSTB20090050C90] Panda S. Nayak S. K. Campo B. Walker J. R. Hogenesch J. B. Jegla T. 2005 Illumination of the melanopsin signaling pathway. Science 307, 600–604 (doi:10.1126/science.1105121) 15681390 10.1126/science.1105121

[RSTB20090050C91] Pandey S. Blanks J. C. Spee C. Jiang M. Fong H. K. 1994 Cytoplasmic retinal localization of an evolutionary homolog of the visual pigments. Exp. Eye Res. 58, 605–613 (doi:10.1006/exer.1994.1055) 7925698 10.1006/exer.1994.1055

[RSTB20090050C92] Peirson S. Foster R. G. 2006 Melanopsin: another way of signaling light. Neuron 49, 331–339 (doi:10.1016/j.neuron.2006.01.006) 16446137 10.1016/j.neuron.2006.01.006

[RSTB20090050C93] Peirson S. N. Bovee-Geurts P. H. Lupi D. Jeffery G. DeGrip W. J. Foster R. G. 2004 Expression of the candidate circadian photopigment melanopsin (*Opn4*) in the mouse retinal pigment epithelium. Brain Res. Mol. Brain Res. 123, 132–135 (doi:10.1016/j.molbrainres.2004.01.007) 15046875 10.1016/j.molbrainres.2004.01.007

[RSTB20090050C94] Peirson S. N. Oster H. Jones S. L. Leitges M. Hankins M. W. Foster R. G. 2007 Microarray analysis and functional genomics identify novel components of melanopsin signaling. Curr. Biol. 17, 1363–1372 (doi:10.1016/j.cub.2007.07.045) 17702581 10.1016/j.cub.2007.07.045

[RSTB20090050C95] Pepe I. M. 2001 Recent advances in our understanding of rhodopsin and phototransduction. Prog. Retin Eye Res. 20, 733–759 (doi:10.1016/S1350-9462(01)00013-1) 11587916 10.1016/s1350-9462(01)00013-1

[RSTB20090050C96] Philp A. R. Bellingham J. Garcia-Fernandez J. Foster R. G. 2000a A novel rod-like opsin isolated from the extra-retinal photoreceptors of teleost fish. FEBS Lett. 468, 181–188 (doi:10.1016/S0014-5793(00)01217-5) 10692583 10.1016/s0014-5793(00)01217-5

[RSTB20090050C97] Philp A. R. Garcia-Fernandez J. M. Soni B. G. Lucas R. J. Bellingham J. Foster R. G. 2000b Vertebrate ancient (VA) opsin and extraretinal photoreception in the Atlantic salmon (*Salmo salar*). J. Exp. Biol. 203, 1925–1936 10821749 10.1242/jeb.203.12.1925

[RSTB20090050C98] Pires S. S. Shand J. Bellingham J. Arrese C. Turton M. Peirson S. Foster R. G. Halford S. 2007 Isolation and characterization of melanopsin (Opn4) from the Australian marsupial Sminthopsis crassicaudata (fat-tailed dunnart). Proc. Biol. Sci. 274, 2791–2799 (doi:10.1098/rspb.2007.0976) 17785267 10.1098/rspb.2007.0976 PMC3227130

[RSTB20090050C99] Pittendrigh C. S. 1993 Temporal organisation: reflections of a Darwinian clock-watcher. Annu. Rev. Physiol. 55, 17–54 (doi:10.1146/annurev.ph.55.030193.000313) 10.1146/annurev.ph.55.030193.000313 8466172

[RSTB20090050C100] Provencio I. Foster R. G. 1995 Circadian rhythms in mice can be regulated by photoreceptors with cone-like characteristics. Brain Res. 694, 183–190 (doi:10.1016/0006-8993(95)00694-L) 8974643 10.1016/0006-8993(95)00694-l

[RSTB20090050C101] Provencio I. Jiang G. De Grip W. J. Hayes W. P. Rollag M. D. 1998 Melanopsin: an opsin in melanophores, brain, and eye. Proc. Natl Acad. Sci. USA 95, 340–345 (doi:10.1073/pnas.95.1.340) 9419377 10.1073/pnas.95.1.340 PMC18217

[RSTB20090050C102] Provencio I. Rodriguez I. R. Jiang G. Hayes W. P. Moreira E. F. Rollag M. D. 2000 A novel human opsin in the inner retina. J. Neurosci. 20, 600–605 10632589 10.1523/JNEUROSCI.20-02-00600.2000 PMC6772411

[RSTB20090050C103] Qiu X. Kumbalasiri T. Carlson S. M. Wong K. Y. Krishna V. Provencio I. Berson D. M. 2005 Induction of photosensitivity by heterologous expression of melanopsin. Nature 433, 745–749 (doi:10.1038/nature03345) 15674243 10.1038/nature03345

[RSTB20090050C104] Rodieck R. W. 1998 In The first steps in seeing Sunderland, MA: Sinauer Associates

[RSTB20090050C105] Roenneberg T. Foster R. G. 1997 Twilight times: light and the circadian system. Photochem. Photobiol. 66, 549–561 (doi:10.1111/j.1751-1097.1997.tb03188.x) 9383985 10.1111/j.1751-1097.1997.tb03188.x

[RSTB20090050C106] Ruby N. F. Brennan T. J. Xie X. Cao V. Franken P. Heller H. C. O'Hara B. F. 2002 Role of melanopsin in circadian responses to light. Science 298, 2211–2213 (doi:10.1126/science.1076701) 12481140 10.1126/science.1076701

[RSTB20090050C107] Saitou N. Nei M. 1987 The neighbor-joining method: a new method for reconstructing phylogenetic trees. Mol. Biol. Evol. 4, 406–425 3447015 10.1093/oxfordjournals.molbev.a040454

[RSTB20090050C108] Sekaran S. Foster R. G. Lucas R. J. Hankins M. W. 2003 Calcium imaging reveals a network of intrinsically light-sensitive inner-retinal neurons. Curr. Biol. 13, 1290–1298 (doi:10.1016/S0960-9822(03)00510-4) 12906788 10.1016/s0960-9822(03)00510-4

[RSTB20090050C109] Sekaran S. Lupi D. Jones S. L. Sheely C. J. Hattar S. Yau K. W. Lucas R. J. Foster R. G. Hankins M. W. 2005 Melanopsin-dependent photoreception provides earliest light detection in the mammalian retina. Curr. Biol. 15, 1099–1107 (doi:10.1016/j.cub.2005.05.053) 15964274 10.1016/j.cub.2005.05.053 PMC4316668

[RSTB20090050C110] Sekaran S. Lall G. S. Ralphs K. L. Wolstenholme A. J. Lucas R. J. Foster R. G. Hankins M. W. 2007 2-Aminoethoxydiphenylborane is an acute inhibitor of directly photosensitive retinal ganglion cell activity in vitro and in vivo. J. Neurosci. 27, 3981–3986 (doi:10.1523/JNEUROSCI.4716-06.2007) 17428972 10.1523/JNEUROSCI.4716-06.2007 PMC6672550

[RSTB20090050C111] Selinger H. H. 1962 Direct action of light in naturally pigmented muscle fibres. I. Action spectrum for contraction in eel iris sphincter. J. Gen. Physiol. 46, 277–283 13992712 10.1085/jgp.46.2.333 PMC2195268

[RSTB20090050C112] Shand J. Foster R. G. 1999 The extraretinal photoreceptors of non-mammalian vertebrates. In Adaptive Mechanisms in the Ecology of Vision (eds Archer S. N. Djamgoz M. B. A. Loew E. R. Partridge J. C. Vallerga S.), pp. 197–222 Dordrecht, The Netherlands: Kluwer Academic Publishers

[RSTB20090050C113] Shichida Y. Matsuyama T. 2009 Evolution of opsins and phototransduction. Phil. Trans. R. Soc. B 364, 2881–2895 (doi:10.1098/rstb.2009.0051) 19720651 10.1098/rstb.2009.0051 PMC2781858

[RSTB20090050C114] Silver R. Witkovsky P. Horvath P. Alones V. Barnstable C. J. Lehman M. N. 1988 Coexpression of opsin- and VIP-like-immunoreactivity in CSF-contacting neurons of the avian brain. Cell Tissue. Res. 253, 189–198 (doi:10.1007/BF00221754) 2970894 10.1007/BF00221754

[RSTB20090050C115] Solessio E. Engbretson G. A. 1993 Antagonistic chromatic mechanisms in photoreceptors of the parietal eye of lizards. Nature 364, 442–445 (doi:10.1038/364442a0) 8332214 10.1038/364442a0

[RSTB20090050C116] Soni B. G. Foster R. G. 1997 A novel and ancient vertebrate opsin. FEBS Lett. 406, 279–283 (doi:10.1016/S0014-5793(97)00287-1) 9136902 10.1016/s0014-5793(97)00287-1

[RSTB20090050C117] Soni B. Philp A. Knox B. Foster R. 1998 Novel retinal photoreceptors. Nature 394, 27–28 (doi:10.1038/27794) 9665123 10.1038/27794

[RSTB20090050C118] Su C. Y. Luo D. G. Terakita A. Shichida Y. Liao H. W. Kazmi M. A. Sakmar T. P. Yau K. W. 2006 Parietal-eye phototransduction components and their potential evolutionary implications. Science 311, 1617–1621 (doi:10.1126/science.1123802) 16543463 10.1126/science.1123802

[RSTB20090050C119] Sun H. Gilbert D. J. Copeland N. G. Jenkins N. A. Nathans J. 1997 Peropsin, a novel visual pigment-like protein located in the apical microvilli of the retinal pigment epithelium. Proc. Natl Acad. Sci. USA 94, 9893–9898 (doi:10.1073/pnas.94.18.9893) 9275222 10.1073/pnas.94.18.9893 PMC23288

[RSTB20090050C120] Tamura K. Dudley J. Nei M. Kumar S. 2007 MEGA4: Molecular Evolutionary Genetics Analysis (MEGA) software version 4.0. Mol. Biol. Evol. 24, 1596–1599 (doi:10.1093/molbev/msm092) 17488738 10.1093/molbev/msm092

[RSTB20090050C121] Taniguchi Y. Hisatomi O. Yoshida M. Tokunaga F. 2001 Pinopsin expressed in the retinal photoreceptors of a diurnal gecko. FEBS Lett. 496, 69–74 (doi:10.1016/S0014-5793(01)02395-X) 11356185 10.1016/s0014-5793(01)02395-x

[RSTB20090050C122] Tarttelin E. E. Bellingham J. Hankins M. W. Foster R. G. Lucas R. J. 2003 Neuropsin (Opn5): a novel opsin identified in mammalian neural tissue. FEBS Lett. 554, 410–416 (doi:10.1016/S0014-5793(03)01212-2) 14623103 10.1016/s0014-5793(03)01212-2

[RSTB20090050C123] Tu D. C. Batten M. L. Palczewski K. Van Gelder R. N. 2004 Nonvisual photoreception in the chick iris. Science 306, 129–131 (doi:10.1126/science.1101484) 15459395 10.1126/science.1101484

[RSTB20090050C124] van Veen T. Hartwig H. G. Müller K. 1976 Light dependent motor activity and photonegative behavior in the eel (*Anguilla anguilla L*.). J. Comp. Physiol. A 111, 209–219 (doi:10.1007/BF00605532)

[RSTB20090050C125] Vigh-Teichmann I. Korf H. W. Oksche A. Vigh B. 1982 Opsin-immunoreactive outer segments and acetylcholinesterase-positive neurons in the pineal complex of *Phoxinus phoxinus* (Teleostei, Cyprinidae). Cell Tissue Res. 227, 351–369 (doi:10.1007/BF00210891) 6217894 10.1007/BF00210891

[RSTB20090050C126] Vigh-Teichmann I. Korf H. W. Nurnberger F. Oksche A. Vigh B. Olsson R. 1983 Opsin-immunoreactive outer segments in the pineal and parapineal organs of the lamprey (*Lampetra fluviatilis*), the eel (*Anguilla anguilla*), and the rainbow trout (*Salmo gairdneri*). Cell Tissue Res. 230, 289–307 6221801 10.1007/BF00213806

[RSTB20090050C127] Vollrath L. 1981 In The pineal organ Heidelberg, Germany: Springer

[RSTB20090050C128] von Frisch K. 1911 Beitrage zur Physiologie der Pigmentzellen in der Fischhaut. Pfluger's Archv. Gesamte Physiol. Menschen Tiere 138, 319–387 (doi:10.1007/BF01680752)

[RSTB20090050C129] Weber W. 1983 Photosensitivity of chromatophores. Am. Zool. 23, 495–506

[RSTB20090050C130] White J. H. 2008 Identification of a novel asthma susceptibility gene on chromosome 1qter and its functional evaluation. Hum. Mol. Genet. 17, 1890–1903 (doi:10.1093/hmg/ddn087) 18344558 10.1093/hmg/ddn087

[RSTB20090050C131] Whitmore D. Foulkes N. S. Sassone-Corsi P. 2000 Light acts directly on organs and cells in culture to set the vertebrate circadian clock. Nature 404, 87–91 (doi:10.1038/35003589) 10716448 10.1038/35003589

[RSTB20090050C132] Wolken J. J. Mogus M. A. 1979 Extra-ocular photo-sensitivity. Photochem. Photobiol. 29, 189–196 (doi:10.1111/j.1751-1097.1979.tb09281.x)

[RSTB20090050C133] Yokoyama S. Zhang H. 1997 Cloning and characterization of the pineal gland-specific opsin gene of marine lamprey (*Petromyzon marinus*). Gene 202, 89–93 (doi:10.1016/S0378-1119(97)00458-7) 9427550 10.1016/s0378-1119(97)00458-7

[RSTB20090050C134] Yoshikawa T. Okano T. Oishi T. Fukada Y. 1998 A deep brain photoreceptive molecule in the toad hypothalamus. FEBS Lett. 424, 69–72 (doi:10.1016/S0014-5793(98)00139-2) 9537517 10.1016/s0014-5793(98)00139-2

[RSTB20090050C135] Yoshimura T. Ebihara S. 1996 Spectral sensitivity of photoreceptors mediating phase-shifts of circadian rhythms in retinally degenerate CBA/J (rd/rd) and normal CBA/N (+/+) mice. J. Comp. Physiol 178A, 797–802 (doi:10.1007/BF00225828) 8667293 10.1007/BF00225828

[RSTB20090050C136] Young J. Z. 1962 In The life of the vertebrates Oxford, UK: The Clarendon Press

